# Pyrolysis temperature and time of rice husk biochar potentially control ammonia emissions and Chinese cabbage yield from urea-fertilized soils

**DOI:** 10.1038/s41598-024-54307-2

**Published:** 2024-03-08

**Authors:** Yun-Gu Kang, Jin-Hyuk Chun, Yeo-Uk Yun, Jun-Yeong Lee, Jwakyung Sung, Taek-Keun Oh

**Affiliations:** 1 Department of Bio-Environmental Chemistry, Chungnam National University, Daejeon, 34134 South Korea; 2 The Korea Ginseng Inspection Office, National Agricultural Cooperative Federation, Geumsan, 32747 South Korea; 3 Division of Environmentally Friendly Agriculture, Chungcheongnam-do Agricultural Research and Extension Services, Yesan, 32418 South Korea; 4 Department of Crop Science, Chungbuk National University, Cheongju, 28644 South Korea

**Keywords:** Environmental sciences, Environmental monitoring

## Abstract

Current agricultural practices are increasingly favoring the biochar application to sequester carbon, enhance crop growth, and mitigate various environmental pollutants resulting from nitrogen (N) loss. However, since biochar’s characteristics can vary depending on pyrolysis conditions, it is essential to determine the optimal standard, as they can have different effects on soil health. In this study, we categorized rice husk biochars basis on their pH levels and investigated the role of each rice husk biochar in reducing ammonia (NH_3_) emissions and promoting the growth of Chinese cabbage in urea-fertilized fields. The findings of this study revealed that the variation in pyrolysis conditions of rice husk biochars and N rates affected both the NH_3_ emissions and crop growth. The neutral (pH 7.10) biochar exhibited effective NH_3_ volatilization reduction, attributed to its high surface area (6.49 m^2^ g^−1^), outperforming the acidic (pH 6.10) and basic (pH 11.01) biochars, particularly under high N rates (640 kg N ha^−1^). Chinese cabbage yield was highest, reaching 4.00 kg plant^−1^, with the basic biochar application with high N rates. Therefore, the neutral rice husk biochar effectively mitigate the NH_3_ emissions from urea-treated fields, while the agronomic performance of Chinese cabbage enhanced in all biochar amendments.

## Introduction

Given the increasing focus on sustainable ecosystems and eco-friendly agriculture, contemporary agricultural practices encounter several challenges^1^. These challenges include the necessity to reduce the use of chemical fertilizers and pesticides, adopt minimal tillage techniques, incorporate organic amendments (e.g., organic fertilizer, manure compost, and biochar), and effectively manage nutrient lossess^1,2^. Specifically, the continuous and excessive application of nutrients, such as nitrogen (N) and phosphorus (P), through chemical fertilization can result in various environmental contaminations^2^. These contaminations involve the release of particulate matter (PM), greenhouse gases (GHGs), eutrophication, and algal bloom in both the atmosphere and aquatic ecosystems^3–5^. Ammonia (NH_3_) volatilization stands out as a prominent source of N losses and contributes to the formation of secondary PM (PM_2.5_) and nitrous oxide (N_2_O)^2,4,5^. Furthermore, NH_3_ emissions has detrimental effects on air quality^5^, human health^4,6^, and the Earth’s radiative balance^2^. These pollutants further exacerbate the impacts of global warming and climate change^5^.

Numerous studies have been dedicated to the development of sustainable and eco-friendly agricultural practices with the aim of reducing N losses, particularly NH_3_, while simultaneously enhancing crop productivity^7–10^. These practices encompass a range of approaches, including the application of natural urease inhibitors^2,3^, the introduction of elemental sulfur and polymers^9^, the use of organic fertilizer^4^, and the incorporation of biochar amendments^7,9,10^. Biochar, a carbon-rich material, is obtained through the pyrolysis of agricultural residues, biomass, and organic waste ingredients under relatively high temperatures and oxygen-limited conditions^7,9–12^. It has garnered attention for its distinctive characteristics, such as carbon (C) sequestration^7,13^, promotion of plant growth^14,15^, enhancement of soil pH^4^, optimization of soil health^12^, provision of a habitat for microorganisms^13^, and the adsorption of heavy metals and nutrient contents^4,16^. Furthermore, biochar has the ability to absorb organic N, ammonium ions (NH_4_^+^), and gaseous NH_3_ through its functional groups and microspores, resulting in reduced N losses^15^. These properties of biochar are evident in the reduced N loss observed in agricultural soils treated with N fertilizers in the presence of biochar^17^. Unfortunately, many experiments have focused on the combined effects of several substitutes, such as urease inhibitor^2,18,19^, wood vinegar^20^, zeolite^21^, and compost^22,23^, or have explored the influence of biochar’s formulation^24^ and feedstock sources^25^ on NH_3_ emissions in agricultural soil. This variation in results may be attributed to the diverse characteristics of biochar produced under different pyrolysis conditions. Therefore, further studies are necessary to assess the efficiency of NH_3_ emission reduction by biochar, taking into account biochar characteristics such as pH and surface area, which are related to N adsorption capacity.

We hypothesize that (1) higher pH levels in rice husk biochar might increase soil pH, hypothetically affecting NH_3_ mitigation efficiency, and (2) excessive N rates could disturb Chinese cabbage yield. To assess these hypotheses, this study evaluated NH_3_ volatilization and crop yield in a Chinese cabbage field treated with different rates of N fertilizer and three types of rice husk biochar classified based on their pH levels. The rice husk biochars were categorized as acidic (AB, pH 6.10), neutral (NB, pH 7.10), and basic (BB, pH 11.01), while N rates applied as urea were designed as N_0.5_ (160 kg N ha^−1^), N_1.0_ (320 kg N ha^−1^, the recommended N rate), and N_2.0_ (640 kg N ha^−1^), respectively. Results revealed that both NH_3_ mitigation efficiency by the rice husk biochars and Chinese cabbage yield increased with rising N rates from 160 to 640 kg N ha^−1^. Interestingly, NH_3_ emissions from N fertilization were lowest in the soil treated with NB, which had the highest surface area compared to AB and BB. Due to the conflicting influences between BB’s alkali effect and urea’s pH-reducing impact, the N_0.5_ treatment exhibited higher soil pH than the N_2.0_ treatment, and soil chemical properties except for soil pH did not reach negative levels in the N_2.0_ treatment. These unexpected findings suggest that the NH_3_ mitigation rate primarily depended on the rice husk biochar’s surface area rather than their pH values. Moreover, there was no negative effect in crop yield caused by excessive N supply owing to higher initial soil pH and the increased NH_3_ emissions.

## Results

### Pyrolysis conditions affect the characteristics of the rice husk biochar

Table 1 presents the chemical properties of the rice husk biochars and their corresponding pyrolysis conditions. The variations in pyrolysis temperature and time had a significant impact on the chemical properties of the rice husk biochar. The pH of the rice husk biochar exhibited a sharp increase as the pyrolysis temperature and time were raised from 400 to 600 °C and from 15 to 30 min, respectively. In contrast, the electrical conductivity (EC) values of AB, NB, and BB gradually decreased with the increase in their pyrolysis conditions. The surface area (SA) of the rice husk biochars was the highest in NB at 6.49 m^2^ g^−1^, while AB and BB were observed at 2.55 and 5.30 m^2^ g^−1^, respectively. The total carbon (TC) content of BB was significantly higher at 54.90% compared to 41.30% of AB and 44.10% of NB, while the total nitrogen (TN) content did not show a statistically significant difference among AB, NB, and BB. Conversely, the total hydrogen (TH) and total oxygen (TO) contents decreased with the increase in pyrolysis conditions and were the highest values in AB at 5.39 and 34.61%, respectively. Inorganic contents of the rice husk biochar gradually increased with the increased in pyrolysis conditions. The H:C and O:C ratio, which represent the aromaticity and polarity of the rice husk biochar, were higher at lower temperatures and shorter times.

**Table 1:** Chemical characteristics of rice husk biochar produced from different pyrolysis conditions.

Samples	Pyrolysis conditions		pH (1:10, H_2_O)	EC (dS m^−1^)	Surface area (m^2^ g^−1^)	TC (%)	TN (%)	TH (%)	TO (%)	TP (%)	CaO (%)	K_2_O (%)	MgO (%)	Na_2_O (%)	H:C ratio (%)	O:C ratio (%)
	Temp. (°C)	Time (min)														
AB	330	15	6.10 ± 0.01^c^	11.49 ± 1.62^a^	2.55 ± 0.01^c^	41.30 ± 0.01^b^	0.40 ± 0.02^a^	5.39 ± 0.11^a^	34.61 ± 0.59^a^	0.14 ± 0.03^a^	0.08 ± 0.02^a^	0.36 ± 0.12^b^	0.04 ± 0.02^a^	0.03 ± 0.01^a^	1.55 ± 0.06^a^	0.63 ± 0.32^a^
NB	400	15	7.10 ± 0.02^b^	9.50 ± 0.83^b^	6.49 ± 0.03^a^	44.10 ± 0.02^b^	0.40 ± 0.02^a^	5.32 ± 0.03^a^	32.50 ± 1.33^a^	0.16 ± 0.02^a^	0.09 ± 0.03^a^	0.47 ± 0.07^b^	0.04 ± 0.01^a^	0.03 ± 0.01^a^	1.44 ± 0.02^b^	0.55 ± 0.21^a^
BB	600	30	11.01 ± 0.05^a^	6.59 ± 0.13^c^	5.30 ± 0.05^b^	54.90 ± 0.19^a^	0.60 ± 0.01^a^	2.11 ± 0.03^b^	5.88 ± 1.98^b^	0.21 ± 0.01^a^	0.16 ± 0.05^a^	0.78 ± 0.09^a^	0.07 ± 0.03^a^	0.04 ± 0.01^a^	0.46 ± 0.08^c^	0.08 ± 0.09^b^
p-value			**	***	**	**	***	**	***	*	*	**	***	***	**	***

*AB* acidic (pH 6.1) rice husk biochar; *NB* neutral (pH 7.1) rice husk biochar; *BB* basic (pH 11.0) rice husk biochar; *EC* electrical conductivity, *TC* total carbon, *TN* total nitrogen, *TH* total hydrogen, *TO* total oxygen, *T-P* total phosphorus.

**Figure 1:**
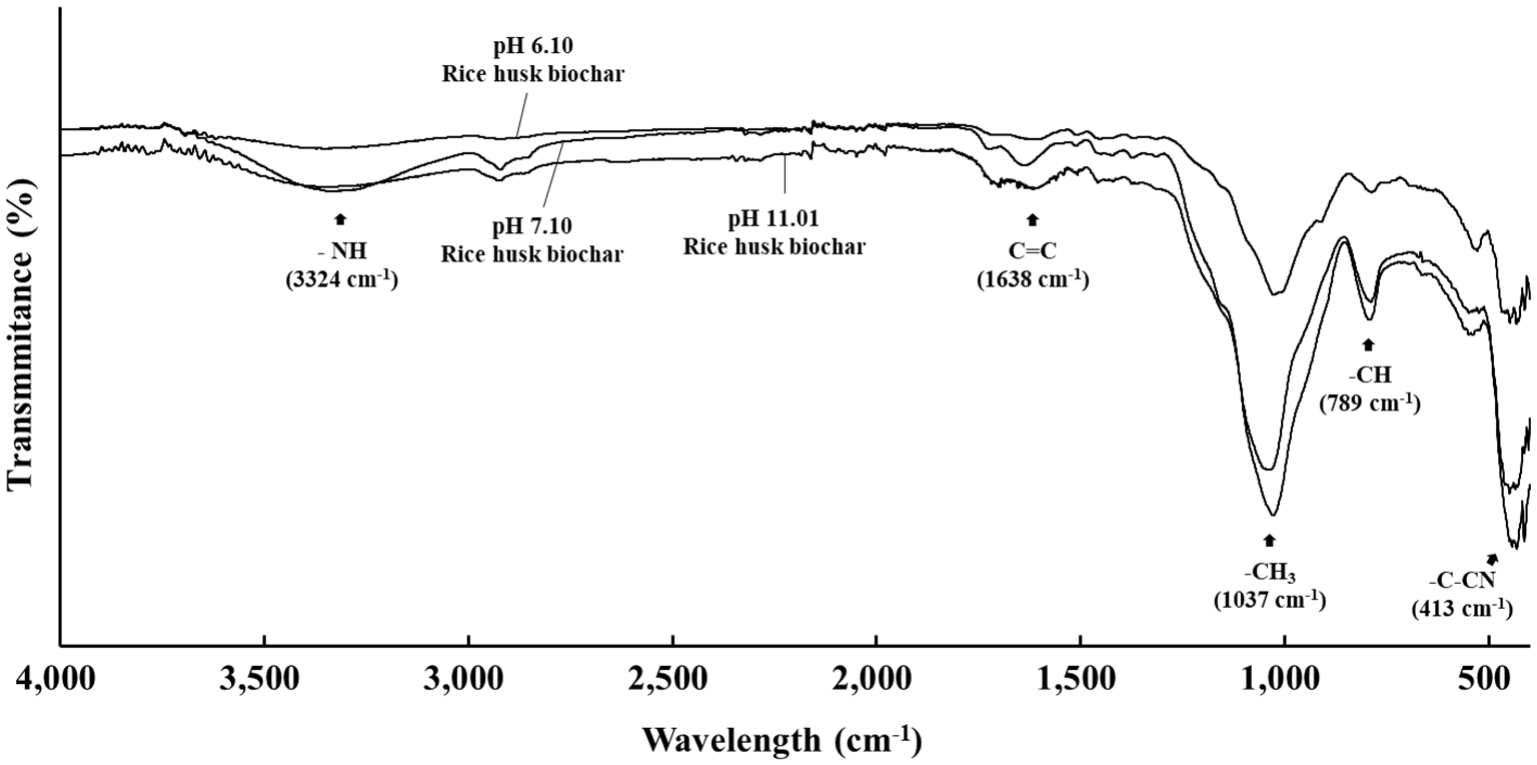
FT-IR spectrum of rice husk biochars categorized by their pH values.

The results of the analysis of functional groups on the surface of the rice husk biochar using Fourier transform infrared spectroscopy (FT-IR) were presented in Fig. 1. The secondary amide group, indicated by the –NH bond in the range of 3300–3325 cm^−1^, was observed in NB and BB but not in AB. The C=C, –CH_3_, and –C–CN bonds in the range of 1640–1660 cm^−1^, 1000–1050 cm^−1^, and 400–420 cm^−1^, respectively, were strongly formed with the increased pyrolysis conditions.

### Ammonia volatilization reduce effectively by the neutral rice husk biochar

**Figure 2:**
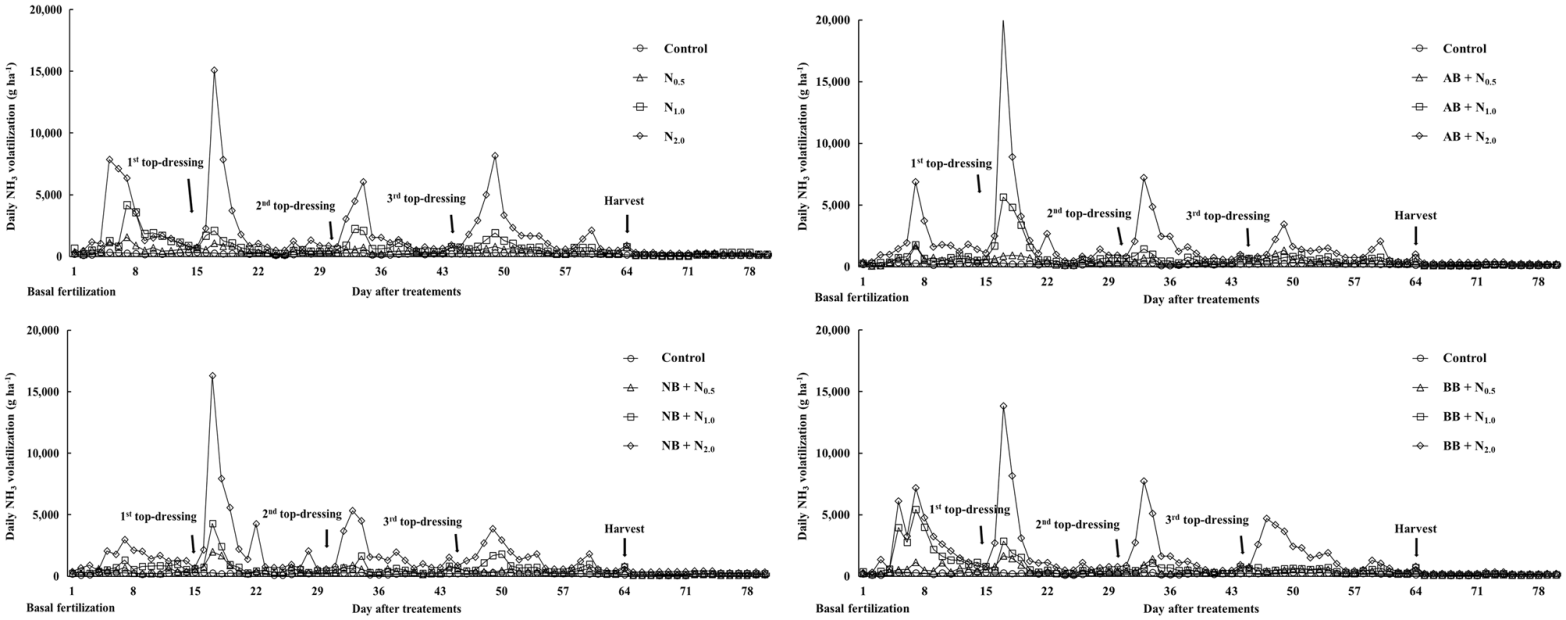
Daily NH_3_ volatilization effected by different nitrogen rates and three types of rice husk biochar during the Chinese cabbage cropping period. N_0.5_, N_1.0_, and N_2.0_ exhibited nitrogen application rates equivalent to 160 kg N ha^−1^, 320 kg N ha^−1^, and 640 kg N ha^−1^, respectively, while AB, NB, and BB donated the acidic (pH 6.1), neutral (pH 7.1), and basic (pH 11.0) rice husk biochars.

**Figure 3:**
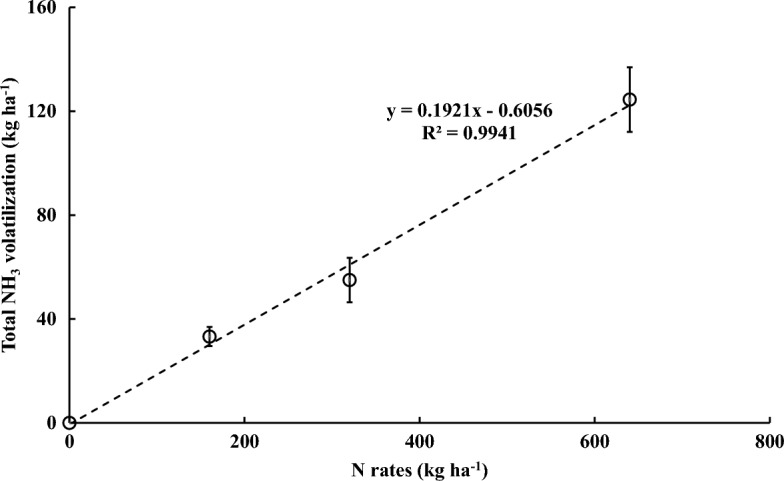
Correlation between nitrogen rates and total NH_3_ volatilization.

Figure 2 displays the daily NH_3_ volatilization resulting from different N rates and rice husk biochar amendments. The NH_3_ emissions peaked within 7 days after N application, with the first top-dressing fertilization leading to the maximum NH_3_ release compared to the basal and other top-dressing fertilizations. Furthermore, the NH_3_ peaks were higher with increasing the N rates (Fig. 3). The AB + N_2.0_ treatment recorded the highest peak value at 20,127.94 g ha^−1^ day^−1^ (20.13 kg ha^−1^ day^−1^), while the NB + N_2.0_ and BB + N_2.0_ treatments reached 16,300.87 (16.30 kg ha^−1^ day^−1^) and 13,847.16 g ha^−1^ day^−1^ (13.85 kg ha^−1^ day^−1^), respectively. After reaching the highest peak, the NH_3_ volatilization sharply decreased and became similar to the control with non-N fertilization.

**Figure 4:**
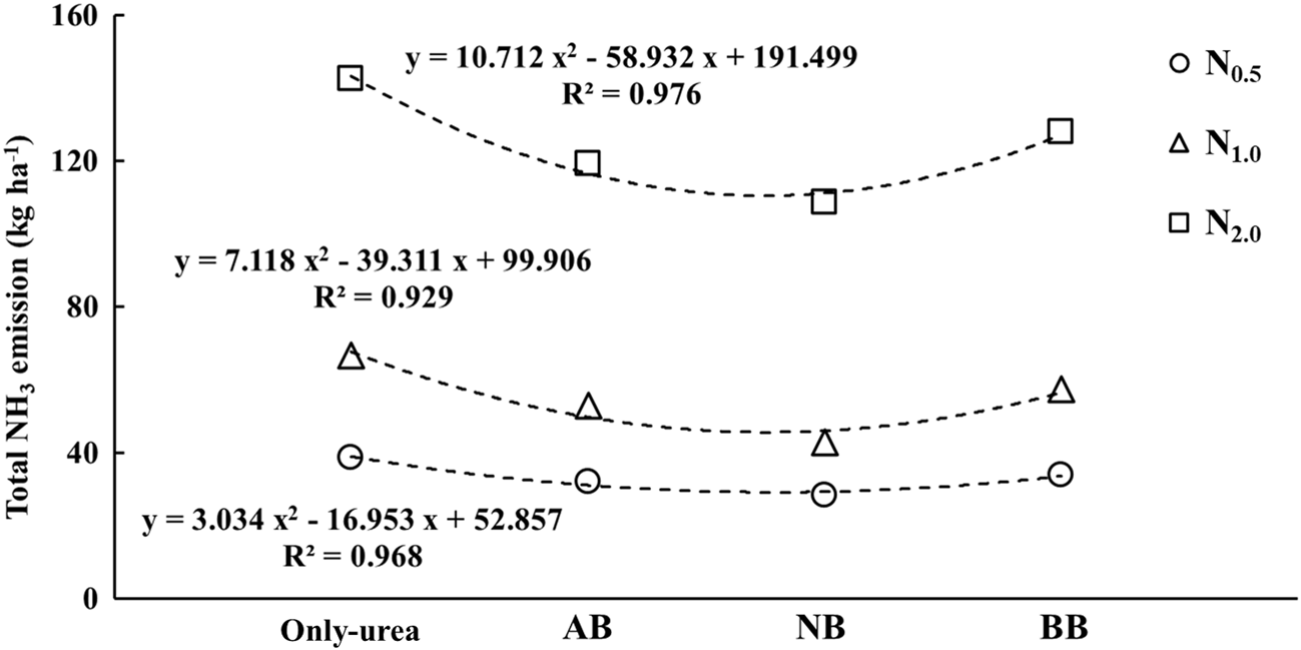
Total NH_3_ emissions effected by three types of rice husk biochar and different nitrogen rates. N_0.5_, N_1.0_, and N_2.0_ exhibited nitrogen application rates equivalent to 160 kg N ha^−1^, 320 kg N ha^−1^, and 640 kg N ha^−1^, respectively, while AB, NB, and BB donated the acidic (pH 6.1), neutral (pH 7.1), and basic (pH 11.0) rice husk biochars.

Figure 4 illustrates the total NH_3_ emissions during the Chinese cabbage cropping season. The total NH_3_ emissions were influenced by the N rates, and the reduction efficiency on NH_3_ emission varied depending on the pH of the rice husk biochar (Supplementary Table S1). Cumulative NH_3_ emissions were the lowest in NB treatments, such as NB + N_0.5_, NB + N_1.0_, and NB + N_2.0_, at 28.42, 42.99, and 108.54 kg ha^−1^, respectively. In contrast, the only-urea treatments (i.e., N_0.5_, N_1.0_, and N_2.0_) had the highest values at 38.64, 66.70, and 142.42 kg ha^−1^, respectively. In comparison to the soil treated with basic rice husk biochar, the soil treated with acidic rice husk biochar exhibited lower NH_3_ emissions, resulting in reductions of total NH_3_ emissions by 6, 8, and 7% with varying N rates (N_0.5_, N_1.0_, and N_2.0_). Moreover, the reductions in the total NH_3_ emissions attributed to the rice husk biochar amendments were more pronounced with higher N rates, from N_0.5_ to N_2.0_, effectively mitigating the N losses. The highest reduction efficiency by the rice husk biochar was shown in the NB + N_1.0_ treatment at 36% compared to the N_1.0_ treatment.

### Growth of Chinese cabbage increases the N rates and the pH of the rice husk biochar

Table 3 presents the growth characteristics of Chinese cabbage influenced by the varying N rates and rice husk biochar amendments. The BB + N_2.0_ treatment achieved the highest fresh weight at 4.00 kg plant^−1^, while the N_2.0_, AB + N_2.0_, and NB + N_2.0_ treatments yielded 3.40, 3.63, and 3.89 kg plant^−1^, respectively. Additionally, fresh weight increased with the rising N rates and the pH of the rice husk biochar from N_0.5_ to N_2.0_ and from pH 6.10 to pH 11.01, respectively (Supplementary Table S2). However, the moisture contents of each treatment did not exhibit statistical significant difference. Head height and width were the highest in the BB + N_2.0_ treatment, measuring 25.87 and 16.70 cm, respectively. Head growth increased with the increase in the N rates and the pH of the rice husk biochar. Furthermore, leaf length and width were higher with increasing the N rates and the pH of rice husk biochar, but statistically significant differences were observed only in the control treatment. The chlorophyll and TN content of Chinese cabbage were the highest in NB + N_0.5_ and AB + N_1.0_, with SPAD values of 35.19 and a TN content of 3.71%, respectively, although they exhibited a non-specific trend.

### Soil chemical properties change the N rates and the properties of the rice husk biochar

The soil chemical properties were influenced by both the N rates and the pH of the rice husk biochar (Table 2). Soil pH decreased as the N rates increased from 160 kg N ha^−1^ (N_0.5_) to 640 kg N ha^−1^ (N_2.0_), while soil EC increased. Furthermore, among the rice husk biochar amendments, soil pH increased with the rise in the pH of rice husk biochar. The EC values of the soil treated with AB, NB, and BB were lower than those of treatments with only urea (i.e., N_0.5_, N_1.0_, and N_2.0_). The highest soil pH and EC were observed at pH 7.48 in BB + N_0.5_ and 1.26 dS m^−1^ in N_2.0_, respectively. The rice husk biochar amendments effectively increased soil TC and TN contents compared to treatments with only urea. For instance, the co-application of BB and N_2.0_ yielded the highest TC content at 2.36%, while the individual treatments of N_0.5_, N_1.0_, and N_2.0_ decreased from the initial soil pH value of 0.71% to 0.61, 0.66, and 0.64%, respectively. In contrast, soil TN content increased with N fertilization, although no statistically significant difference was observed. Soil available nitrogen (Avail. N) content increased with the rice husk biochar amendment, with NB effectively increasing the Avail. N content under the same N rates conditions. In contrast, there were no significant differences observed in available phosphorus (Avail. P) content of N-treated soil (e.g., N_0.5_, N_1.0_, AB + N_0.5_, NB + N_1.0_, and BB + N_2.0_). The highest Avail. P content was recorded in BB + N_1.0_ at 125.05 mg kg^−1^, while the Avail. P content of initial soil and control was 94.10 and 89.26 mg kg^−1^, respectively. After rice husk biochar amendment and N fertilization, the content of exchangeable cations, such as Ca^2+^, K^+^, Mg^2+^, and Na^+^, increased, but no statistically significant difference was observed.

**Table 2:** Changes in soil chemical properties affected by different nitrogen rates and biochar amendments.

Treatments		pH (1:5, H_2_O)	EC (dS m^−1^)	TC (%)	TN (%)	OM (%)	Avail. N (mg kg^−1^)	Avail. P (mg kg^−1^)	Exchangeable cations			
									Ca^2+^ (cmol_c_ kg^−1^)	K^+^ (cmol_c_ kg^−1^)	Mg^2+^ (cmol_c_ kg^−1^)	Na^+^ (cmol_c_ kg^−1^)
Initial soil		7.00 ± 0.20^c^	0.35 ± 0.05^d^	0.71 ± 0.19^d^	0.11 ± 0.05^b^	1.22 ± 0.33^d^	24.77 ± 0.76f.	94.10 ± 21.08^b^	4.50 ± 0.21^ab^	0.21 ± 0.03^c^	1.24 ± 0.11^b^	0.19 ± 0.00^a^
Control		7.45 ± 0.16^a^	0.40 ± 0.06^d^	0.44 ± 0.23^e^	0.04 ± 0.01^c^	0.76 ± 0.40^e^	48.11 ± 9.34^e^	89.26 ± 4.73^b^	4.48 ± 0.14^ab^	0.23 ± 0.04^c^	1.20 ± 0.10^b^	0.19 ± 0.01^a^
N_0.5_		7.16 ± 0.36^bc^	0.75 ± 0.39^b^	0.61 ± 0.19^d^	0.16 ± 0.02^a^	1.05 ± 0.33^d^	74.51 ± 11.07^d^	113.38 ± 3.90^a^	4.59 ± 0.10^ab^	0.23 ± 0.04^c^	1.46 ± 0.05^a^	0.18 ± 0.02^a^
N_1.0_		6.80 ± 0.17^ cd^	1.12 ± 0.29^a^	0.66 ± 0.13^d^	0.19 ± 0.03^a^	1.14 ± 0.22^d^	88.75 ± 17.32^c^	117.40 ± 17.30^a^	4.62 ± 0.05^ab^	0.27 ± 0.05^bc^	1.50 ± 0.01^a^	0.20 ± 0.01^a^
N_2.0_		6.59 ± 0.18^e^	1.26 ± 0.49^a^	0.64 ± 0.18^d^	0.21 ± 0.03^a^	1.10 ± 0.31^d^	104.34 ± 12.46^bc^	112.74 ± 9.97^a^	4.69 ± 0.30^b^	0.29 ± 0.08^bc^	1.48 ± 0.18^a^	0.21 ± 0.02^a^
AB	N_0.5_	7.19 ± 0.18^bc^	0.52 ± 0.09^c^	1.03 ± 0.12^c^	0.17 ± 0.02^a^	1.78 ± 0.21^c^	94.51 ± 16.07^ cd^	120.69 ± 19.97^a^	4.60 ± 0.13^ab^	0.23 ± 0.05^c^	1.54 ± 0.12^a^	0.21 ± 0.05^a^
	N_1.0_	7.19 ± 0.49^bc^	0.58 ± 0.34^c^	1.19 ± 0.06^c^	0.18 ± 0.02^a^	2.05 ± 0.10^c^	101.33 ± 14.02^bc^	119.69 ± 19.63^a^	4.63 ± 1.05^ab^	0.33 ± 0.03^b^	1.56 ± 0.09^a^	0.22 ± 0.04^a^
	N_2.0_	7.12 ± 0.43^bc^	1.09 ± 0.38^a^	1.14 ± 0.17^c^	0.20 ± 0.03^a^	1.97 ± 0.29^c^	124.98 ± 25.78^a^	115.08 ± 11.34^a^	4.90 ± 0.37^a^	0.48 ± 0.04^a^	1.59 ± 0.08^a^	0.22 ± 0.02^a^
NB	N_0.5_	7.26 ± 0.22^b^	0.64 ± 0.37^c^	1.64 ± 0.37^b^	0.17 ± 0.01^a^	2.83 ± 0.64^b^	97.90 ± 18.08^c^	118.01 ± 17.98^a^	4.54 ± 0.57^ab^	0.31 ± 0.09^b^	1.52 ± 0.03^a^	0.20 ± 0.01^a^
	N_1.0_	7.14 ± 0.36^bc^	0.69 ± 0.34^c^	1.78 ± 0.68^b^	0.18 ± 0.01^a^	3.07 ± 1.17^b^	115.80 ± 14.48^b^	120.56 ± 11.74^a^	4.66 ± 0.21^ab^	0.37 ± 0.03^ab^	1.57 ± 0.25^a^	0.22 ± 0.04^a^
	N_2.0_	7.13 ± 0.69^bc^	1.16 ± 0.40^a^	1.62 ± 0.49^b^	0.21 ± 0.01^a^	2.79 ± 0.84^b^	143.31 ± 23.44^a^	123.44 ± 5.80^a^	4.96 ± 0.52^a^	0.44 ± 0.09^a^	1.56 ± 0.08^a^	0.25 ± 0.09^a^
BB	N_0.5_	7.48 ± 0.21^a^	0.67 ± 0.16^c^	2.09 ± 0.58^a^	0.20 ± 0.01^a^	3.60 ± 1.00^a^	93.87 ± 17.01^ cd^	113.42 ± 6.22^a^	4.33 ± 0.07^b^	0.32 ± 0.04^b^	1.55 ± 0.02^a^	0.21 ± 0.02^a^
	N_1.0_	7.44 ± 0.22^a^	0.88 ± 0.04^b^	2.06 ± 0.53^a^	0.21 ± 0.01^a^	3.55 ± 0.91^a^	104.33 ± 12.46^bc^	125.05 ± 9.92^a^	4.61 ± 0.34^ab^	0.41 ± 0.07^a^	1.58 ± 0.11^a^	0.26 ± 0.08^a^
	N_2.0_	7.24 ± 0.64^ab^	1.18 ± 0.24^a^	2.36 ± 0.45^a^	0.23 ± 0.01^a^	4.07 ± 0.76^a^	138.61 ± 19.82^a^	117.17 ± 34.87^a^	4.97 ± 0.55^a^	0.43 ± 0.06^a^	1.57 ± 0.08^a^	0.20 ± 0.02^a^
p-value		*	**	***	**	***	*	***	**	*	***	***

N_0.5_, 160 kg N ha^−1^; N_1.0_, 320 kg N ha^−1^; N_2.0_, 640 kg N ha^−1^.

*AB* acidic (pH 6.1) rice husk biochar, *NB* neutral (pH 7.1) rice husk biochar, *BB* basic (pH 11.0) rice husk biochar, *EC* electrical conductivity, *TC* total carbon, *TN* total nitrogen, *OM* organic matter, *Avail. N* available nitrogen, *Avail. P* available phosphorus.

*, **, and *** are used to indicate statistically significant differences at the p < 0.05, p < 0.01, and p < 0.001, respectively.

^a^^–f^Each value with different letters within a column are significantly different from each other as determined by Duncan’s multiple range test (p < 0.05).

## Discussion

Numerous prior studies have consistently shown that an increase in pyrolysis temperature results in heightened parameters such as pH, surface area, cation exchange capacity, and carbon content of biochar^26,27^. Particularly, the escalation in biochar pH predominantly arises from carbonate formation and the elevation in inorganic alkali contents^28,29^. Furthermore, the pH of biochar increases owing to presence of ash content and oxygen functional groups^30^. However, the composition of cellulose and hemicellulose in plant-based ingredients occurs at relatively low temperature (between 200 and 300 °C) and generate various organic acids and phenolic substances that decrease the pH of the material^30^. This implies that biochar produced at lower temperature might exhibit a lower pH compared to the initial raw material. On the other hand, the TH, and TO contents of rice husk biochars decreased with the increasing the pyrolysis conditions, resulting in a sequential reduction of H:C and O:C ratio. These findings indicate that pyrolysis conditions play a crucial role in regulating the element composition of the rice husk biochar, thereby influencing its quality in terms of stability and aromaticity. The stability and aromaticity of rice husk biochar are reflected in the H:C and O:C ratio, respectively, with lower values considered superior. Previous studies have reported that higher pyrolysis conditions lead to an increase in the proportion of non-volatile compounds, particularly aromatic substances^31,32^. As the content of aromatic substances rises, the fixed carbon content and non-volatile compounds in rice husk biochar increase, contributing to the enhancement of its stability and aromaticity^32^. Furthermore, the aforementioned parameters were also decreased by the TC content of rice husk biochar, showing a positive (+) correlation with pyrolysis conditions.

The NH_3_ emissions from agricultural soils are potentially depended on several factors such as the presence of soil amendments, the pH and moisture content of agricultural soil, method of nitrogen fertilizer application, and various agricultural practice (e.g., tillage, irrigation duration, and soil mulching)^4,33,34^. The PCA results by Liu et al.^35^ were indicated that NH_3_ volatilization varied in a descending order as follow: soil type, N source, soil pH, soil environmental conditions (e.g., temperature and moisture content). The application of biochar can adjust soil pH and enhance soil drainage, thereby improving the soil environment, which may influence NH_3_ emissions^10,36^. Previous studies reported results indicating that biochar amendments promote the NH_3_ emissions from agricultural soil owing to their alkali effects, which increase the soil pH^36–38^. In particular, high soil pH leads to higher rates of NH_3_ volatilization because it raises the NH_3_ concentrations dissolved in soil moisture^39^. Furthermore, another study documented that total NH_3_ emissions increased by 10 to 71% with higher application rates of biochar^40^. These studies primarily focused on changes in soil pH influenced by the pH of biochar, and the NH_3_ losses were found to be more pronounced in soil pH levels between 7 and 8^41^. To effectively manage the NH_3_ emissions from agricultural land, it is necessary to maintain the soil pH below 7.0.

Conversely, several previous studies, which yielded conflicting results compared to the aforementioned studies, indicated that biochar amendments can effectively reduce the NH_3_ volatilization from urea-treated soil under various conditions^42,43^. They demonstrated that the functional groups on the surface, adsorption ability, and cation exchange capacity of biochar contribute to decreasing NH_3_ emissions from N-fertilized agricultural soils^44–46^. In this study, the soil amended with AB, NB, and BB exhibited lower NH_3_ emissions compared to the solely urea-treated soil, which had the lowest soil pH values. These findings suggest that the reduction efficiency of NH_3_ emissions by rice husk biochars, attributed to their functional groups, microspores, and adsorption ability, outweighs the increase in NH_3_ emissions associated with elevated soil pH values. Furthermore, the reduction efficiency of rice husk biochars varied based on their pH, with BB amendment exhibiting higher NH_3_ emissions compared to AB and NB amendments. As the pyrolysis conditions increased, the functional groups of BB decreased (Fig. 1), indicating a potential decrease in the NH_3_ reduction efficiency of BB. This reduction could lead to a relatively higher NH_3_ emissions, particularly when compared to AB or NB amendments, emphasizing the impact of pyrolysis conditions on the ammonia reduction efficiency of the biochar.

The application of rice husk biochars has been proven to enhance the growth and N uptake of Chinese cabbage, as shown in Table 3. This is supported by several previous studies that have examined the relationship between plant growth and biochar amendment^47^. Crop growth is primarily influenced by soil health, and biochar amendments are one of the factors that improve soil properties, fertility, and quality^48^. For instance, Munoz et al.^47^ illustrated that biochar amendments can reduce both soil bulk density and particle density, while Peake et al.^48^ demonstrated that the application of biochar improves soil compaction by more than 10%. Additionally, biochar application enhances soil fertility as it supplies essential elements such as N, P, K, Ca, Mg, Fe, and Si^48^. The findings of this study also support the notion that soil nutrient contents (e.g., Avail. N and Avail. P) were increased by rice husk biochar amendments (Table 2). The application of rice husk biochars increased the soil Avail. N content by capturing gaseous NH_3_ and NH_4_^+^ through their functional groups. Nitrogen fixation by biochar was achieved through the surface characteristics of the biochar, primarily characterized by a negative charge^10^. The biochar absorbed N in cationic from (i.e., NH_4_^+^), and it exhibited superiority with a large surface area. In this study, the application of NB (6.49 m^2^ g^−1^), which had a larger surface area compared to AB (2.55 m^2^ g^−1^) and BB (5.30 m^2^ g^−1^), resulted in the highest Avail. N content in the N-fertilized soil under the same N rates condition (Table 2). However, since the ionic bond between the biochar surface and the cationic form of N needs to be disconnected for N uptake by plants, the fixed N was not immediately utilized by plants in the short term. These reasons supported our findings, which demonstrated the highest fresh weight of Chinese cabbage in the short-term cultivation experiment with BB amendment, not NB amendment, attributed to the higher soil OM content. Although, not showing statistically significant differences among AB, NB, and BB amendments, the NB application may still improve soil fertility over the long term, resulting in better crop yields.

**Table 3:** Growth characteristics of Chinese cabbage affected by the pH of rice husk biochar and different nitrogen rates.

Treatments		Fresh weight (kg plant^−1^)	Moisture content (%)	Head		Leaf			TN (%)
				Height (cm)	Width (cm)	Length (cm)	Width (cm)	Chlorophyll content (SPAD)	
Control		0.70 ± 0.14f.	90.51 ± 2.21^a^	15.10 ± 2.43^b^	6.93 ± 1.43^c^	24.91 ± 0.68^c^	15.34 ± 0.17^b^	19.32 ± 3.01^c^	2.62 ± 0.08^b^
N_0.5_		2.41 ± 0.10^e^	91.20 ± 0.81^a^	22.63 ± 2.80^a^	12.30 ± 0.36^b^	33.26 ± 0.57^ab^	22.59 ± 0.81^a^	25.15 ± 1.11^b^	2.51 ± 0.19^b^
N_1.0_		3.08 ± 0.73^d^	92.80 ± 2.12^a^	23.53 ± 4.18^a^	13.73 ± 0.56^b^	33.20 ± 2.16^ab^	23.02 ± 2.54^a^	29.95 ± 4.90^ab^	2.52 ± 0.26^b^
N_2.0_		3.40 ± 0.25^c^	93.11 ± 0.62^a^	24.97 ± 2.25^a^	15.33 ± 1.33^a^	34.81 ± 1.15^ab^	24.90 ± 0.16^a^	30.51 ± 1.41^ab^	2.55 ± 0.03^b^
AB	N_0.5_	2.63 ± 0.24^e^	92.11 ± 1.52^a^	22.47 ± 0.70^a^	12.27 ± 0.59^b^	31.52 ± 0.93^b^	22.93 ± 0.37^a^	32.37 ± 0.73^a^	3.68 ± 0.01^a^
	N_1.0_	3.17 ± 0.09^ cd^	92.44 ± 0.82^a^	23.03 ± 0.83^a^	13.73 ± 0.67^b^	33.23 ± 1.81^ab^	23.08 ± 1.49^a^	33.71 ± 1.82^a^	3.71 ± 0.04^a^
	N_2.0_	3.63 ± 0.23^ab^	92.08 ± 1.26^a^	24.53 ± 1.50^a^	15.70 ± 0.87^a^	33.91 ± 1.30^ab^	24.98 ± 2.10^a^	30.37 ± 1.44^ab^	3.70 ± 0.02^a^
NB	N_0.5_	2.67 ± 0.18^e^	91.16 ± 0.87^a^	22.40 ± 2.10^a^	12.77 ± 0.78^b^	32.32 ± 1.50^ab^	22.38 ± 0.73^a^	35.19 ± 5.76^a^	3.69 ± 0.05^a^
	N_1.0_	3.30 ± 0.37^c^	91.90 ± 1.32^a^	24.07 ± 2.19^a^	13.87 ± 1.96^b^	33.27 ± 2.40^ab^	24.09 ± 2.96^a^	34.76 ± 3.08^a^	3.69 ± 0.06^a^
	N_2.0_	3.89 ± 0.24^a^	91.32 ± 0.69^a^	25.63 ± 2.00^a^	16.13 ± 0.83^a^	35.28 ± 0.97^a^	24.65 ± 1.25^a^	32.43 ± 4.56^a^	3.65 ± 0.04^a^
BB	N_0.5_	2.73 ± 0.11^e^	90.09 ± 0.75^a^	23.20 ± 2.08^a^	12.63 ± 1.45^b^	32.63 ± 1.30^ab^	22.21 ± 0.95^a^	31.28 ± 2.73^ab^	3.67 ± 0.11^a^
	N_1.0_	3.52 ± 0.17^b^	91.42 ± 1.15^a^	24.93 ± 1.81^a^	15.17 ± 0.21^a^	35.72 ± 0.67^a^	24.37 ± 0.68^a^	30.55 ± 1.30^ab^	3.65 ± 0.14^a^
	N_2.0_	4.00 ± 0.54^a^	91.34 ± 0.47^a^	25.87 ± 0.42^a^	16.70 ± 0.87^a^	36.08 ± 0.75^a^	24.49 ± 1.21^a^	34.84 ± 3.10^a^	3.66 ± 0.04^a^
p-value		**	***	**	**	***	**	**	***

N_0.5_, 160 kg N ha^−1^; N_1.0_, 320 kg N ha^−1^; N_2.0_, 640 kg N ha^−1^.

*AB* acidic (pH 6.1) rice husk biochar, *NB* neutral (pH 7.1) rice husk biochar, *BB* basic (pH 11.0) rice husk biochar, *TN* total nitrogen.

*, **, and *** are used to indicate statistically significant differences at the p < 0.05, p < 0.01, and p < 0.001, respectively.

^a^^–f^Each value with different letters within a column are significantly different from each other as determined by Duncan’s multiple range test (p < 0.05).

## Conclusions

This study demonstrates the significant impact of rice husk biochar amendments in mitigating the NH_3_ emissions during the Chinese cabbage cropping period. The NH_3_ emissions resulting from chemical fertilization in agricultural soil decreased in the presence of rice husk biochar. Notably, the neutral (pH 7.10) rice husk biochar amendment exhibited the most substantial reduction in the NH_3_ volatilization compared to the acidic (pH 6.10) and basic (pH 11.01) rice husk biochars. Furthermore, biochar amendments improved the Chinese cabbage yield, and this improvement was more pronounced with an increase in the pH of rice husk biochar. The highest agronomic performance of Chinese cabbage was observed in the basic rice husk biochar treatment with the 640 kg N ha^−1^ (N_2.0_). Therefore, the application of neutral rice husk biochar can effectively reduce the NH_3_ emissions from N-fertilized agricultural soil, while basic rice husk biochar leads to the highest agronomic performance and yield of Chinese cabbage.

## Materials and methods

### Experimental site

**Figure 5:**
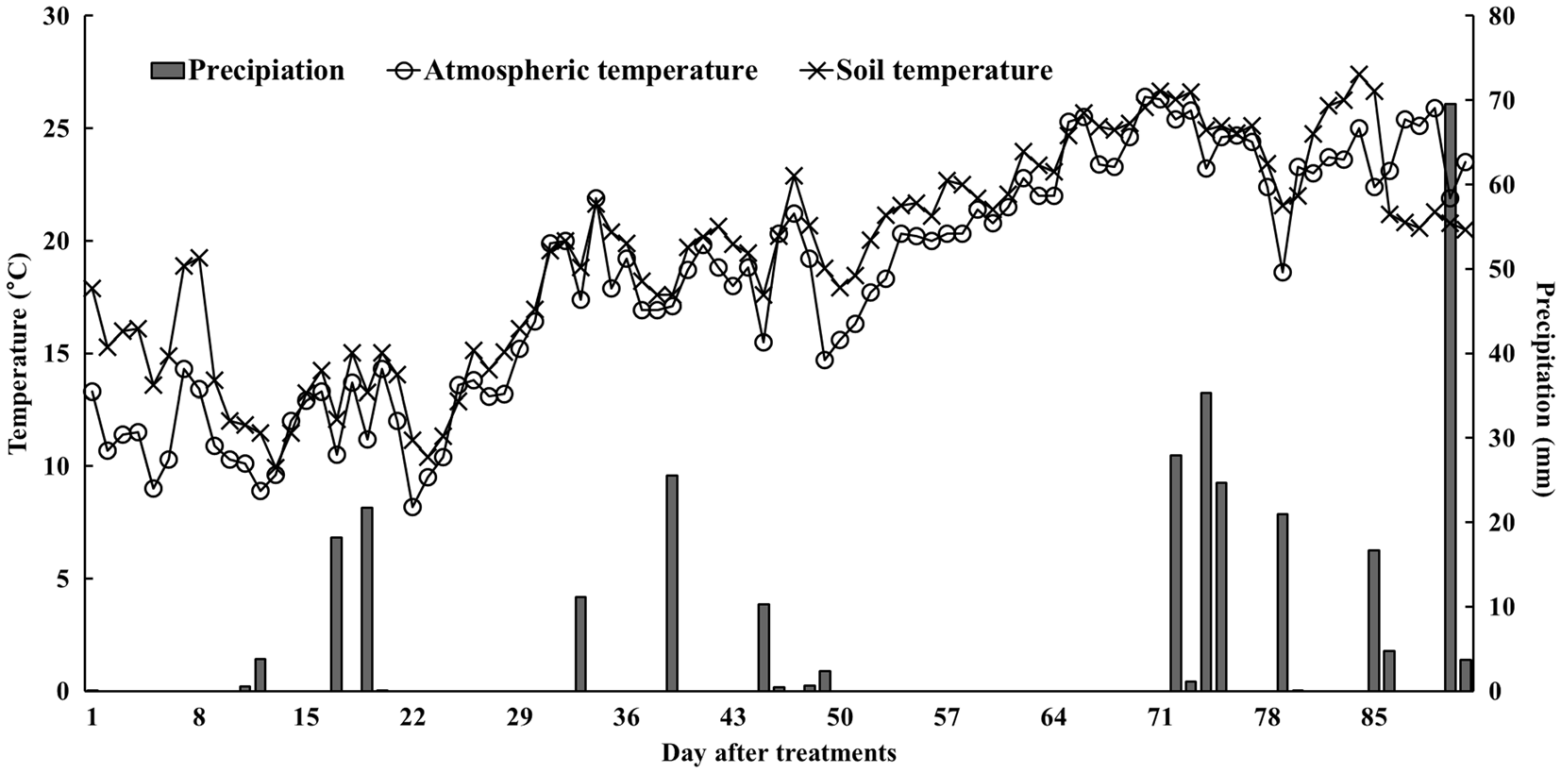
The meteorological data during the Chinses cabbage cultivation.

This study was conducted at the experimental field located in Chungnam National University, Daejeon, South Korea (35° 14′ 12.8″ N, 139° 7′ 0.5″ E). The experimental area experiences a humid continental and subtropical climate, both of which are influenced by the East Asian Monsoon^4^. During the summer season, which typically begins in June or July, the area receives high precipitation and is occasionally affected by typhoons. Detailed meteorological conditions during the cultivation period are presented in Fig. 5. The experimental field had been conventionally used for cultivating Chinese cabbage for approximately 5-year. The soil in the experimental field is classified as sandy loam, consisting of 12.8% clay, 41.4% silt, and 45.8% sand, and it belongs to the Inceptisols order.

### Preparation of rice husk biochar

The rice husk biochars were prepared under different pyrolysis conditions using an electrical furnace (1100 °C Box Furnace, Thermo Scientific Inc., Waltham, Massachusetts, USA). Initially, rice husks sourced from rice paddy at Chungnam National University underwent thorough washing with deionized water to eliminate several impurities (e.g., bird poop, insect corpse, soil, and crop residue). Subsequently, the damp rice husks were stored in a glass greenhouse for 2-week to remove their moisture content. Following this, the dried samples were placed in a stainless-steel barrel (Ø 260 × 140 mm) with and aluminum packing, and subjected to pyrolysis using an electrical furnace. In this study, the aluminum packing was used to block the oxygen (O_2_) inflow. Finally, the rice husk biochars were categorized based on their pH values, specifically pH 6.1 (AB), pH 7.1 (NB), and pH 11.0 (BB). AB was produced at 350 °C for 15 min, while NB and BB were manufactured at 450 °C for 15 min and 600 °C for 30 min, respectively. The selected pyrolysis conditions were established based on prior studies^15^, that delineated the chemical properties of rice husk biochar under varying pyrolysis conditions, and preliminary experiment (Supplementary Table S3). In this study, AB exhibited the relatively minor differences from NB, likely attributed to the initial pH of the rice husk (pH 6.27). However, BB showed discernible differences from NB with increasing pyrolysis conditions. Therefore, we extended the pyrolysis time for BB from 15 to 30 min to observed the effect of stark pH differences.

### Cultivation experiment

The cultivation experiment spanned a duration of 80 days, from April 12 to Jun 30, 2021, and followed a randomized complete block design with three replications. The ‘Chunkwang’ variety of Chinese cabbage (*Brassica rapa* L.) was sown in each plot, covering an area of 2.5 m × 3.0 m (7.5 m^2^) with two rows. This study comprised thirteen treatments, including the following: control (non-fertilization), N fertilizer applied at recommended rate (320 kg N ha^−1^, N_1.0_), N fertilizer applied at half the recommended rate (160 kg N ha^−1^, N_0.5_), and N fertilizer applied at double the recommended rate (640 kg N ha^−1^, N_2.0_), as well as combined applications of the rice husk biochars (i.e., AB, NB, and BB) with N fertilizers (i.e., AB + N_1.0_, AB + N_0.5_, AB + N_2.0_, NB + N_1.0_, NB + N_0.5_, NB + N_2.0_, BB + N_1.0_, BB + N_0.5_, and BB + N_2.0_). The rice husk biochars were applied to the agricultural soil at a rate of 1% (w w^−1^), which was recommended by previous studies^49^, and a mechanical tiller was used to incorporate the rice husk biochars with the soil. Before transplanting, 78 kg P_2_O_5_ ha^−1^, and 60 kg K_2_O ha^−1^, in the form of fused phosphate and potassium chloride, respectively, were applied as basal fertilizer. Additionally, 46 kg K_2_O ha^−1^ of potassium chloride was applied at 15, 30, and 45 days after transplanting. Similarly, 55, 110, and 220 kg N ha^−1^, in the form of urea, were applied as basal fertilizer, with 35, 70, and 140 kg N ha^−1^ applied in three installments during the cultivation period. The plots were irrigated every 2 days and after each fertilizer application to prevent water stress.

### Ammonia measurement and analysis

The measurement of daily and total NH_3_ emissions during the Chinese cabbage cultivation period was conducted using a static chamber made of acrylic material (h: 30 × Ø: 12 cm, 0.011 m^2^)^24^. To capture the released NH_3_, a sponge soaked in a glycerol-phosphoric acid solution was placed inside the chamber for 24 h. Collection of gaseous NH_3_ was performed daily throughout the Chinese cabbage cultivation period, and after harvest, it was conducted twice a week until NH_3_ volatilization resulting from N fertilization was no longer observed. The collected NH_3_ samples were subsequently extracted using an excess of 2 M potassium chloride solution and quantified using a UV/Vis-spectrophotometer (Genesys 50, Thermo Scientific Inc., Waltham, Massachusetts, USA) following the Indophenol Blue method. Furthermore, another sponge was placed at the top end of the chamber before tightly sealing it. This top sponge served to isolate and absorb air or foreign substances, preventing their interference with the measurements. The daily and total NH_3_ emissions during the Chinese cabbage cultivation period were calculated using the following equations^4,24^.

1 $$ Daily\,NH_{3}\,emission = \frac{{\left( {C \times V} \right)}}{{\left( {t \times A} \right)}} $$

2 $$Total\,{NH}_{3}\,emission= \sum_{i=0}^{n}({N}_{i} \times {D}_{i})$$

In Eq. (1), C represents the NH_4_^+^ concentration in sponge (mg L^−1^), V denotes the volume of NH_4_^+^ solution obtained by sponge squeeze (L), t indicates the time to capture gaseous NH_3_ samples (day), and A is the surface area of chamber (0.011 m^2^). In Eq. (2), N_i_ represents the rate of daily NH_3_ emissions in the *i*th sampling interval, *D*_*i*_ denotes the number of days in the *i*th sampling interval, and *n* represents the number of sampling intervals.

### Soil, biochar, and plant analysis

Soil sample analysis involved the selection of ten random sampling points within each treatment. Soil texture was determined using the hydrometer method. Soil pH and EC were measured in soil slurry, where 1 g of soil was mixed with 5 mL of distilled water, using a pH and EC meter (ORION™ Versa Star Pro™, Thermo Scientific Inc., Waltham, Massachusetts, USA). The TC and TN contents were analyzed using an elemental analyzer (TruSpec Micro, Leco, Michigan, USA), while the OM content was calculated based on the TC content. The Avail. P and Avail. N contents were determined using a UV/Vis-spectrophotometer following the Lancaster method (for Avail. P content), Indophenol Blue method (for NH_4_^+^ content), and Brucine method (for NO_3_^−^ content), respectively. Additionally, the Avail. N content was calculated as the sum of NH_4_^+^ and NO_3_^−^ contents. Soil exchangeable cations were extracted using a neutral 1 M ammonium acetate solution and analyzed using a ICP-OES (ICAP 7000series ICP spectrometer, Thermo Scientific Inc., Waltham, Massachusetts, USA).

The pH of EC of the rice husk biochars were measured in a biochar slurry, where 1 g of biochar was mixed with 10 mL of distilled water, using a pH and EC meter. The BET surface area of the rice husk biochars was determined using a surface area analyzer (ASAP 2420, Micromeritics Inc., Norcross, Georgia, USA). Surface area was assessed using a N gas-adsorption method, and the sorption curves of N gas were analyzed to determine the biochar’s surface area. The TC, TN, TH, and TO contents were analyzed by an elemental analyzer. The TP content was determined using the vanadate molybdate method with a UV/Vis-spectrophotometer. The inorganic contents (i.e., K_2_O, CaO, MgO, and Na_2_O) were analyzed using a FT-IR (Spectrum Two, Perkin Elmer, Waltham, Massachusetts, USA).

The growth parameters of Chinese cabbage were assessed as follows: fresh weight, moisture content, head height, head width, leaf length, leaf width, and chlorophyll content. Fresh weight was measured after harvest. Head height and width were estimated by measuring the half of Chinese cabbage, after measuring the length and width of the top three leaves with a ruler. Chlorophyll content was determined using a chlorophyll meter (SPAD-502 plus, Konica Minolta, Tokyo, Japan).

### Statistical analysis

Each dataset was subjected to statistical analysis using multivariate analysis of variance (MANOVA) followed by Duncan’s multiple range test to determine significant differences at a significant level (p) < 0.05. The statistical analysis was performed using the statistical software SPSS version 4.10.6 (SPSS Inc., Chicago, State of Illinois, USA).

### Ethics approval and consent to participate

The seeds of Chinese cabbage were obtained from Sakata Korea (Seoul, South Korea). This study was conducted by complying with the Agricultural Life Resource Management Guidelines of Rural Development Administration, South Korea, the IUCN Policy Statement on Research Involving Species at Risk of Extinction, and the Convention on the Trade in Endangered Species of Wild Fauna and Flora.

### Supplementary Information

Supplementary Tables.

## Data Availability

All data generated or analyzed during this study are included in this published article [and its supplementary information files].

## References

[1] Pineiro V (2020). A scoping review on incentives for adoption of sustainable agricultural practices and their outcomes. Nat. Sustain..

[2] Dawar K (2021). Biochar and urease inhibitor mitigate NH_3_ and N_2_O emissions and improve wheat yield in a urea fertilized alkaline soil. Sci. Rep..

[3] Dawar K (2021). Effects of the nitrification inhibitor nitrapyrin and the plant growth regulator gibberellic acid on yield-scale nitrous oxide emission in maize fields under hot climatic conditions. Pedosphere.

[4] Kang YG (2022). Influence of individual and co-application of organic and inorganic fertilizer on NH_3_ volatilization and soil quality. J. King Saud Univ. Sci..

[5] Saylor, R., Myles, L., Sibble, D., Caldwell, J. & Xing, J. Recent trends in gas-phase ammonia and PM_2.5_ ammonium in the Southeast United States. *J. Air Waste Manage. Assoc.* **65**, 347–357. 10.1080/10962247.2014.992554 (2015). 10.1080/10962247.2014.992554 25947130

[6] Pope CA, Dockery DW (2006). Health effects of fine particulate air pollution: Lines that connect. J. Air Waste Manage. Assoc..

[7] Chu C (2023). Biochar application can mitigate NH_3_ volatilization in acidic forest and upland soils but stimulates gaseous N losses in flooded acidic paddy soil. Sci. Total Environ..

[8] Ma Z (2019). Mitigation of ammonia volatilization and nitrate leaching via loss control urea triggered H-bond forces. Sci. Rep..

[9] He T (2018). Effects of application of inhibitors and biochar to fertilizer on gaseous nitrogen emissions from an intensively managed wheat field. Sci. Total Environ..

[10] Kang, Y. G. et al. Effects of varying rates of nitrogen and biochar pH on NH_3_ emissions and agronomic performance of Chinese cabbage (Brassica rapa ssp. pekinensis). *Agron.* **12**, 61. 10.3390/agronomy12010061 (2022).

[11] Lin X (2023). Biochar application increases biological nitrogen fixation in soybean with improved soil properties in an Ultisol. J. Soil Sci. Plant Nutr..

[12] Feng Y (2022). How does biochar aging affect NH_3_ volatilization and GHGs emissions from agricultural soil?. Environ. Pollut..

[13] Sheng Y, Zhu L (2018). Biochar alters microbial community and carbon sequestration potential across different soil pH. Sci. Total Environ..

[14] Diatta AA, Fike JH, Battaglia ML, Galbraith JM, Baig MB (2020). Effects of biochar on soil fertility and crop productivity in arid regions: A review. Arab. J. Geosci..

[15] Kang YG (2023). Effect of pyrolysis conditions on chemical properties of carbonized rice husks for efficient NH_4_^+^ adsorption. Appl. Biol. Chem..

[16] Glaser B, Lehr V (2019). Biochar effects on phosphorus availability in agricultural soils: A meta-analysis. Sci. Rep..

[17] Nelissen V, Rutting T, Huygens D, Ruysschaert G, Boeckx P (2015). Temporal evolution of biochar’s impact on soil nitrogen processes—a 15N tracing study. Gcb Bioenergy.

[18] Lan T (2022). Biological nitrification inhibitor co-application with urease inhibitor or biochar yield different synergistic interaction effects on NH_3_ volatilization, N leaching, and N use efficiency in a calcareous soil under rice cropping. Environ. Pollut..

[19] He T (2022). Combined biochar and double inhibitor application offsets NH_3_ and N_2_O emissions and mitigates N leaching in paddy fields. Environ. Pollut..

[20] Sun H (2020). Response of ammonia volatilization from rice paddy soil to application of wood vinegar alone or combined with biochar. Chemosphere.

[21] Ali A (2022). Mitigating ammonia and greenhouse gaseous emission from arable land by co-application of zeolite and biochar. Front. Plant Sci..

[22] Alarefee HA, Ishak CF, Othman R, Karam DS (2023). Effectiveness of mixing poultry litter compost with rice husk biochar in mitigating ammonia volatilization and carbon dioxide emission. J. Environ. Manag..

[23] Wu D (2019). Biochar combined with vermicompost increases crop production while reducing ammonia and nitrous oxide emissions from a paddy soil. Pedosphere.

[24] Egyir M, Luyima D, Kim SH, Oh TK (2022). Effects of modified and nitrogen-enriched biochars on ammonia emissions and crop yields under a field environment. Water Air Soil Pollut..

[25] Ji M (2022). Effects of different feedstocks-based biochar on soil remediation: A review. Environ. Pollut..

[26] Tomczyk A, Sokolowska Z, Boguta P (2020). Biochar physicochemical properties: pyrolysis temperature and feedstock kind effects. Rev. Environ. Sci..

[27] Adebajo, S. O. *et al.* Impacts of rice-husk biochar on soil microbial biomass and agronomic performances of tomato (*Solanum lycopersicum* L.). *Sci. Rep.* **12**, 1787. 10.1038/s41598-022-05757-z (2022). 10.1038/s41598-022-05757-z PMC8810918 35110620

[28] Ding Y (2016). Biochar to improve soil fertility. A review. Agron. Sustain. Dev..

[29] Al-Wabel MI, Al-Omran A, El-Naggar AH, Nadeem M, Usman AR (2013). Pyrolysis temperature induced changes in characteristics and chemical composition of biochar produced from conocarpus wastes. Bioresour. Technol..

[30] Yu H, Zhang Z, Li Z, Chen D (2014). Characteristics of tar formation during cellulose, hemicellulose and lignin gasification. Fuel.

[31] Ortiz LR (2020). Influence of pyrolysis temperature and bio-waste composition on biochar characteristics. Renew. Energy.

[32] Peng X, Ye LL, Wang CH, Zhou H, Sun B (2011). Temperature- and duration-dependent rice straw-derived biochar: Characteristics and its effects on soil properties of an Ultisol in southern China. Soil Tilage Res..

[33] Klimczyk M, Siczek A, Schimmelpfennig L (2021). Improving the efficiency of urea-based fertilization leading to reduction in ammonia emission. Sci. Total Environ..

[34] Jiang Y, Deng A, Bloszies S, Huang S, Zhang W (2017). Nonlinear response of soil ammonia emissions to fertilizer nitrogen. Bio. Fertil. Soils.

[35] Liu G, Li Y, Migliaccio KW, Ouyang Y, Alva AK (2011). Identification of factors most important for ammonia emissions from fertilized soils for potato production using principal component analysis. J. Sustain. Watershed. Sci. Manag..

[36] Ma BL (2010). On-farming assessment of the amount and timing of nitrogen fertilizer on ammonia volatilization. Agron. J..

[37] Cabera ML, Kelly TR, Pancorbo OC, Merka WC, Thompson SA (1994). Ammonia volatilization and carbon dioxide emission from poultry litter: Effects of fractionation and storage time. Commun. Soil Sci. Plant Anal..

[38] Singh G, Arya SK (2021). A review on management of rice straw by use of cleaner technologies: Abundant opportunities and expectations for Indian farming. J. Clean. Prod..

[39] Jones, C. A., Koeing, R. T., Ellsworth, J. W., Brown, B. D. & Jackson, G. D. Management of urea fertilizer to minimize volatilization. *MSU Extension* **EB173**, 1–12 (2007).

[40] Feng Y (2017). Bio-char applied at an appropriate rate can avoid increasing NH_3_ volatilization dramatically in rice paddy soil. Chemosphere.

[41] Potter C, Klooster S, Krauter C (2003). Regional modeling of ammonia emissions from native soil sources in California. Earth Interact..

[42] Uddin S (2021). Ammonia fluxes and emission factors under an intensively managed wetland rice ecosystem. Environ. Sci. Process Impacts.

[43] Mandal S (2018). The effect of biochar feedstock, pyrolysis temperature, and application rate on the reduction of ammonia volatilization from biochar-amended soil. Sci. Total Environ..

[44] Sha Z, Li Q, Lv T, Misselbrook T, Liu X (2019). Response of ammonia volatilization to biochar addition: A meta-analysis. Sci. Total Environ..

[45] Yu H (2019). Biochar amendment improves crop production in problem soils: A review. J. Environ. Manag..

[46] Fang J, Zhan L, Ok YS, Gao B (2018). Minireview of potential applications of hydrochar derived from hydrothermal carbonization of biomass. J. Ind. Eng. Chem..

[47] Munoz, C., Gongora, S. & Zagal, E. Use of biochar as a soil amendment: A brief review. *Chil. J. Agric. Anim. Sci.* **32**, 37–47. http://revistas.udec.cl/index.php/chjaas/article/view/6181 (2016).

[48] Peake LR, Reid BJ, Tang X (2014). Quantifying the influence of biochar on the physical and hydrological properties of dissimilar soils. Geoderma.

[49] Brtnicky M (2021). A critical review of the possible adverse effects of biochar in the soil environment. Sci. Total Environ..

