# Supplementary material for: Pyrolysis temperature and time of rice husk biochar potentially control ammonia emissions and Chinese cabbage yield from urea-fertilized soils
Source: Sci Rep. 2024 Mar 8;14:5692. doi: 10.1038/s41598-024-54307-2 (PMC10920921; doi:10.1038/s41598-024-54307-2)
Supplement: Supplementary file 1 — Supplementary Tables. [file 41598_2024_54307_MOESM1_ESM.docx]

Supplementary table S1. Summary of Pearson’s correlation analysis among total NH_3_ emission, N rates, and chemical properties of rice husk biochars

|  | **pH** | **EC** | **Surface area** | **TC** | **TN** | **TH** | **TO** | **TP** | **CaO** | **K_2_O** | **MgO** | **Na_2_O** | **H:C ratio** | **O:C ratio** | **N rates** | **NH_3_** |
| --- | --- | --- | --- | --- | --- | --- | --- | --- | --- | --- | --- | --- | --- | --- | --- | --- |
| **pH** | 1.00^***^ |  |  |  |  |  |  |  |  |  |  |  |  |  |  |  |
| **EC** | -0.98^***^ | 1.00^***^ |  |  |  |  |  |  |  |  |  |  |  |  |  |  |
| **Surface area** | +0.41^*^ | -0.60^**^ | 1.00^***^ |  |  |  |  |  |  |  |  |  |  |  |  |  |
| **TC** | +0.99^***^ | -0.98^***^ | +0.41^**^ | 1.00^***^ |  |  |  |  |  |  |  |  |  |  |  |  |
| **TN** | +0.98^***^ | -0.92^***^ | +0.22^**^ | +0.98^***^ | 1.00^***^ |  |  |  |  |  |  |  |  |  |  |  |
| **TH** | -0.99^***^ | +0.92^***^ | -0.24^**^ | -0.98^***^ | -0.99^***^ | 1.00^***^ |  |  |  |  |  |  |  |  |  |  |
| **TO** | -0.99^***^ | +0.94^***^ | -0.29^**^ | -0.99^***^ | -0.99^***^ | +0.99^***^ | 1.00^***^ |  |  |  |  |  |  |  |  |  |
| **TP** | +0.99^***^ | -0.99^***^ | +0.48^*^ | +0.99^***^ | +0.96^***^ | -0.97^***^ | -0.98^***^ | 1.00^***^ |  |  |  |  |  |  |  |  |
| **CaO** | +0.99^***^ | -0.96^***^ | +0.33^**^ | +0.99^***^ | +0.99^***^ | -0.99^***^ | -0.99^***^ | +0.99^***^ | 1.00^***^ |  |  |  |  |  |  |  |
| **K_2_O** | +0.99^***^ | -0.99^***^ | +0.46^***^ | +0.99^***^ | +0.97^***^ | -0.97^***^ | -0.98^***^ | +0.99^***^ | +0.99^***^ | 1.00^***^ |  |  |  |  |  |  |
| **MgO** | +0.98^***^ | -0.92^***^ | +0.22^*^ | +0.98^***^ | +0.99^***^ | -0.99^***^ | -0.99^***^ | +0.99^***^ | +0.99^***^ | +0.97^***^ | 1.00^***^ |  |  |  |  |  |
| **Na_2_O** | +0.98^***^ | -0.92^***^ | +0.22^**^ | +0.98^***^ | +0.99^***^ | -0.99^***^ | -0.99^***^ | +0.99^***^ | +0.99^***^ | +0.97^***^ | +0.99^***^ | 1.00^***^ |  |  |  |  |
| **H:C ratio** | -0.99^***^ | +0.93^***^ | -0.27^**^ | -0.99^***^ | -0.99^***^ | +0.99^***^ | +0.99^***^ | -0.97^***^ | -0.99^***^ | -0.98^***^ | -0.99^***^ | -0.99^***^ | 1.00^***^ |  |  |  |
| **O:C ratio** | -0.99^***^ | +0.96^***^ | -0.35^***^ | -0.99^***^ | -0.99^***^ | +0.99^***^ | +0.99^***^ | -0.99^***^ | -0.99^***^ | -0.99^***^ | -0.99^***^ | -0.99^***^ | +0.99^***^ | 1.00^***^ |  |  |
| **N rates** | 0.00^***^ | 0.00^***^ | +0.00^***^ | +0.00^***^ | +0.00^***^ | +0.00^***^ | 0.00^***^ | 0.00^***^ | 0.00^***^ | 0.00^***^ | 0.00^***^ | 0.00^***^ | 0.00^***^ | 0.00^***^ | 1.00^***^ |  |
| **NH_3_** | +0.10^**^ | -0.07^***^ | -0.06^**^ | 0.10^**^ | 0.12^**^ | -0.11^***^ | -0.11^*^ | 0.09^*^ | 0.10^*^ | 0.09^**^ | 0.12^***^ | 0.12^**^ | -0.11^**^ | -0.10^*^ | 0.98^***^ | 1.00^***^ |

EC, electrical conductivity; TC, total carbon; TN, total nitrogen; TH, total hydrogen; TO, total oxygen; TP, total phosphorus; NH_3_, ammonia.

*, **, and *** are used to indicate statistically significant differences at the p < 0.05, p < 0.01, and p < 0.001, respectively.

Supplementary table S2. Summary of one-way multivariate analysis of variance (MANOVA)

| Parameter | | Value | F | Hypothesis df | Error df | Significance | Partial Eta Squared |
| --- | --- | --- | --- | --- | --- | --- | --- |
| Intercept | Pillai's Trace | 0.996 | 7426.212 | 6.000 | 186.000 | 0.000 | 0.996 |
|  | Wilks' Lambda | 0.004 | 7426.212 | 6.000 | 186.000 | 0.000 | 0.996 |
|  | Hotelling's Trace | 239.555 | 7426.212 | 6.000 | 186.000 | 0.000 | 0.996 |
|  | Roy's Largest Root | 239.555 | 7426.212 | 6.000 | 186.000 | 0.000 | 0.996 |
| Rice husk biochar pH | Pillai's Trace | 0.549 | 7.017 | 18.000 | 564.000 | 0.000 | 0.183 |
|  | Wilks' Lambda | 0.521 | 7.590 | 18.000 | 526.573 | 0.000 | 0.195 |
|  | Hotelling's Trace | 0.788 | 8.084 | 18.000 | 554.000 | 0.000 | 0.208 |
|  | Roy's Largest Root | 0.558 | 17.487 | 6.000 | 188.000 | 0.000 | 0.358 |
| Intercept | Pillai's Trace | 0.994 | 5276.491 | 6.000 | 186.000 | 0.000 | 0.994 |
|  | Wilks' Lambda | 0.006 | 5276.491 | 6.000 | 186.000 | 0.000 | 0.994 |
|  | Hotelling's Trace | 170.209 | 5276.491 | 6.000 | 186.000 | 0.000 | 0.994 |
|  | Roy's Largest Root | 170.209 | 5276.491 | 6.000 | 186.000 | 0.000 | 0.994 |
| N rates | Pillai's Trace | 0.945 | 14.413 | 18.000 | 564.000 | 0.000 | 0.315 |
|  | Wilks' Lambda | 0.162 | 26.476 | 18.000 | 526.573 | 0.000 | 0.455 |
|  | Hotelling's Trace | 4.544 | 46.613 | 18.000 | 554.000 | 0.000 | 0.602 |
|  | Roy's Largest Root | 4.401 | 137.886 | 6.000 | 188.000 | 0.000 | 0.815 |

df, degree of freedom.

Supplementary table S3. The pH of rice husk biochars based on different pyrolysis conditions

| **Pyrolysis conditions** | |  | **Biochar pH** |
| --- | --- | --- | --- |
| **Temperature** | **Time** |  |  |
| **(°C)** | **(min)** |  | **(1:10, H_2_O)** |
| Original | |  | 6.27±0.03^g^ |
| 300 | 15 |  | 6.18±0.06^g^ |
| 330 | 15 |  | 6.10±0.01^g^ |
|  | 30 |  | 6.67±0.07^f^ |
|  | 60 |  | 7.04±0.12^e^ |
| 400 | 15 |  | 7.10±0.02^e^ |
|  | 30 |  | 8.12±0.11^d^ |
|  | 60 |  | 10.87±0.08^a^ |
| 500 | 15 |  | 7.98±0.08^d^ |
|  | 30 |  | 9.21±0.11^b^ |
|  | 60 |  | 11.06±0.17^a^ |
| 600 | 15 |  | 8.56±0.12^c^ |
|  | 30 |  | 11.01±0.05^a^ |
|  | 60 |  | 11.21±0.12^a^ |

a - g: Each value with different letters within a column are significantly different from each other as determined by Duncan’s multiple range test (p < 0.05).
